# Tumour‐derived exosomes in liver metastasis: A Pandora's box

**DOI:** 10.1111/cpr.13452

**Published:** 2023-03-20

**Authors:** Sini Li, Yan Qu, Lihui Liu, Chao Wang, Li Yuan, Hua Bai, Jie Wang

**Affiliations:** ^1^ National Cancer Center/National Clinical Research Center for Cancer/Cancer Hospital Chinese Academy of Medical Sciences and Peking Union Medical College Beijing China; ^2^ CAMS Key Laboratory of Translational Research on Lung Cancer, State Key Laboratory of Molecular Oncology, Department of Medical Oncology, National Cancer Center/National Clinical Research Center for Cancer/Cancer Hospital Chinese Academy of Medical Sciences and Peking Union Medical College Beijing China

## Abstract

The liver is a common secondary metastasis site of many malignant tumours, such as the colorectum, pancreas, stomach, breast, prostate, and lung cancer. The clinical management of liver metastases is challenging because of their strong heterogeneity, rapid progression, and poor prognosis. Now, exosomes, small membrane vesicles that are 40–160 nm in size, are released by tumour cells, namely, tumour‐derived exosomes (TDEs), and are being increasingly studied because they can retain the original characteristics of tumour cells. Cell–cell communication via TDEs is pivotal for liver pre‐metastatic niche (PMN) formation and liver metastasis; thus, TDEs can provide a theoretical basis to intensively study the potential mechanisms of liver metastasis and new insights into the diagnosis and treatment of liver metastasis. Here, we systematically review current research progress about the roles and possible regulatory mechanisms of TDE cargos in liver metastasis, focusing on the functions of TDEs in liver PMN formation. In addition, we discuss the clinical utility of TDEs in liver metastasis, including TDEs as potential biomarkers, and therapeutic approaches for future research reference in this field.

## Graphical abstract

Exosomes are released by a variety of tumours, including colorectal, pancreatic, gastric, liver, breast, lung, and prostate cancers. Tumour‐derived exosomes (TDEs) reach the liver through the blood and lymphatic system to promote the formation of liver pre‐metastatic niche, thus promoting liver metastasis. Specifically, TDEs induce the liver pre‐metastatic niche formation by promoting the release of inflammatory molecules, promoting epithelial–mesenchymal transition, inducing vascular remodelling, macrophage polarization and mediating immunosuppression.

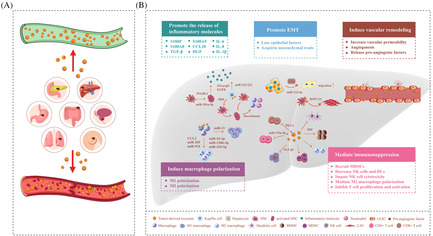

## 1 INTRODUCTION

Metastasis in patients with cancer considerably worsens survival and is the leading cause of cancer‐related deaths.^1^ Because of its distinct structure and biological characteristics, the liver, among other metastatic sites, is the primary metastasis‐permissive site of many malignant tumours, including colorectal, pancreatic, stomach, breast, prostate, and lung cancer.^2^, ^3^, ^4^ The liver is the only organ that has a dual blood supply by the hepatic artery and hepatic portal vein.^5^, ^6^ Liver sinusoidal endothelial cells (LSECs) have a window‐like structure that permits direct entry to tumour cells into the basement membrane.^7^ In addition, the liver has an immune‐tolerant microenvironment that is conducive to foreign tumour cells surviving and growing.^5^, ^8^ Notably, liver metastasis is more common than primary liver tumours, and liver metastasis is often associated with a poor prognosis compared with other sites of metastasis, with about 30%–70% of patients with cancer dying because of liver metastasis.^3^ Unfortunately, the available treatments, including systemic chemotherapy, radiotherapy, immunotherapy, and targeted therapy, are limited and ineffective.^6^, ^9^ Therefore, it is critical and emergent to identify the potential mechanisms of liver metastasis and develop strategies for its prevention and treatment.

The occurrence and development of liver metastasis are closely related to the liver's unique microenvironment, which is composed of cellular components (including liver intrinsic cells, such as hepatocytes, Kupffer cells [KCs], hepatic stellate cells [HSCs], and LSECs; immune cells, such as myeloid‐derived suppressor cells [MDSCs], neutrophils, dendritic cells [DCs], hepatic natural killer [NK] cells, and other lymphocyte subsets) and acellular components (including cell adhesion molecules, cytokines, chemokines, matrix metalloproteinases (MMP), collagen proteins, and growth factors).^2^, ^10^, ^11^ Liver metastasis occurs via a four‐step process involving both cellular and acellular components as follows: (i) the microvascular phase; (ii) the extravascular pre‐angiogenic phase; (iii) the angiogenic phase; and (iv) the growth phase.^4^, ^6^ In recent years, increasing evidence indicates that before tumour cells arrive in the liver, they can initiate and establish a liver microenvironment, that is, a pre‐metastatic niche (PMN), that is conducive to the survival and growth of tumour cells.^12^, ^13^, ^14^ According to Cao, a PMN has the following six characteristics: inflammation, immunosuppression, organotropism, lymph angiogenesis, reprogramming, and angiogenesis or vascular permeability.^15^ A PMN includes extracellular vesicles (EVs), tumour‐derived secreted factors, bone marrow‐derived cells, and the local stromal microenvironment of the host.^15^, ^16^

Exosomes, a subset of EVs, can retain the original characteristics of their parental cells^17^, ^18^ and serve as messengers in intercellular communication by helping transport their cargos from parental cells to recipient cells.^19^ Current studies have shown that tumour‐derived exosomes (TDEs) are an important component of the liver microenvironment and play a pivotal role in establishing liver PMN, promoting, and even determining, liver‐specific metastasis. Furthermore, TDEs can be used as potential biomarkers and may provide a new way to develop therapeutics for cancer with liver metastasis.^14^, ^20^, ^21^ Here, we reviewed exosomes and the potential roles and mechanisms of TDEs in liver metastasis with regard to PMN formation. In addition, we elucidated the most recent advances in the clinical application of TDEs in liver metastasis, including early diagnosis, prognosis, and treatment, to provide novel insights into inhibiting liver metastasis.

## 2 EXOSOME BIOGENESIS, COMPOSITION, AND ISOLATION

**FIGURE 1:**
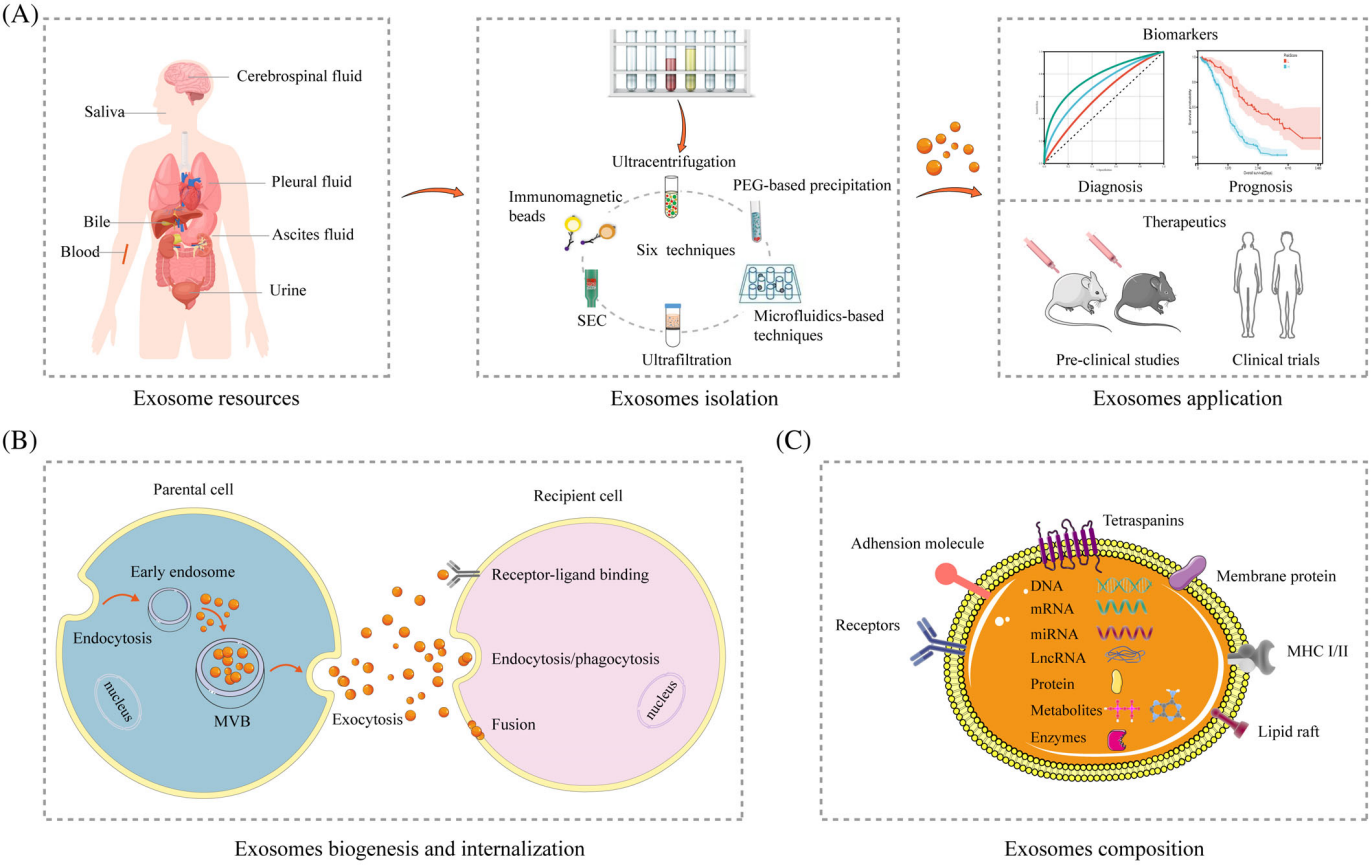
Overview of exosomes. (A) Exosomes resources, isolation methods and applications. (B) The biogenesis and internalization of exosomes. Exosomes are vesicles derived from the fusion of multivesicular bodies with plasma membranes. Cargos of exosomes are delivered to recipient cells through the manner of receptor‐ligand binding, endocytosis/phagocytosis, and fuse directly with the plasma membrane of recipient cells. (C) Typical representative composition of exosomes. The exosome is a round membranous vesicle with a diameter of 40–160 nm and carries parental cell cargos, including lipid bilayers, proteins, nucleic acids (DNA, mRNA, miRNA and lncRNA), enzymes and metabolites.

Exosomes, secreted by most cells, including tumour cells, are ubiquitous in the human body and are found in the blood, urine, saliva, pleural fluid, cerebrospinal fluid, ascites fluid, and other body fluids (Figure 1A).^22^, ^23^ These are about 40–160 nm in diameter, have a round or cup‐shaped structure, and have a density of 1.13–1.19 g/mL.^18^, ^24^ Exosomes have two primary functions, namely, maintaining the homeostasis of the intracellular environment and acting as messengers of intercellular communication, but their specific functions vary depending on their contents and cell sources.^17^, ^18^ The biogenesis process of exosomes is strictly regulated, and it involves the following main steps^17^, ^18^, ^21^, ^25^ (Figure 1B): (i) Endocytosis: The inward invaginations of the cellular plasma membrane contribute to the formation of early endosomes, which later mature into late endosomes. (ii) Formation of multivesicular bodies (MVBs): The maturation of these late endosomes leads to the formation of MVBs and the production of intraluminal vesicles (ILVs) through the acidification and the second invagination of endosomal limiting membranes. The contents of parental cells are, in the interim, incorporated into ILVs. (iii) Exocytosis: Most MVBs contained within the ILVs are degraded after fusing with lysosomes or autophagosomes, but some MVBs fuse with the cellular plasma membrane and release the ILVs into the extracellular environment as mature exosomes. Subsequently, exosomes are internalized by recipient cells through receptor‐ligand binding, endocytosis/phagocytosis, and direct fusion with the plasma membrane of the recipient cells, leading to changes in various biological processes in the recipient cells, including genetic information and the reprogramming of cellular functions.^26^, ^27^

Exosomes contain complex and diverse contents (Figure 1C), including lipid bilayers, proteins, nucleic acids (DNA, mRNA, miRNA, and lncRNA), enzymes, and metabolites that can be transported between cells as a mechanism of communication.^28^, ^29^, ^30^, ^31^ The protein composition of exosomes varies with the origin of cells and tissues. However, a few partially common proteins are found among exosomes of diverse origins, such as MVB‐formation proteins (Alix and TSG 101), membrane transport and fusion proteins (Annexins, Rab GTPases, and flotillin), antigen presentation proteins (HSP60, HSP70, and HSP90), and tetraspanins (CD9, CD63, and CD81); these proteins are used as markers for the identification of exosomes.^18^, ^32^ In addition to these known traditional exosome markers, Hoshino et al. identified 13 novel exosome protein markers, such as ACTB, MSN, STOM, and RAP1B, which contributed to a consensus on exosome markers and provided possibilities for the purification of exosomes.^33^ Today, the contents of exosomes have been extensively studied, particularly proteins and RNA, and several public exosome databases are available, including ExoCarta,^34^ Vesiclepedia,^35^ exoRBase,^36^ EVmiRNA,^37^ and EVpedia.^38^ For instance, the latest data from the Exocarta database show that exosomes include 9769 proteins, 3408 mRNAs, 2838 miRNAs, and 1116 lipids (http://www. Exocarta.org). Despite the extensive research on exosomes, the underlying mechanism by which exosomes selectively package their parental cell‐derived cargos remain unclear, and there are limited studies on the liver metastasis of tumours, an area that needs to be further explored.^25^

The examination of the biological functions of exosomes has largely relied on the ‘purity’ of exosome samples. Therefore, in the past decade, based on the properties of an exosome, numerous exosome isolation technologies have emerged.^39^, ^40^, ^41^, ^42^ Here, we summarize six exosome isolation technologies, all of which have unique advantages and disadvantages (Table 1). In addition to these methods, commercial products are available for exosome isolation that are easy to use, cost‐effective, and can effectively increase the yield of exosomes.^40^, ^43^ Despite the availability of these techniques, the effective acquisition and accurate separation of high‐purity exosomes remain huge challenges owing to the heterogeneity and complexity of exosomes, which is also the primary obstacle for clinical application.^44^, ^45^, ^46^ Therefore, reliable and standard methods must be developed for exosome isolation.

**TABLE 1:** Comparison of six exosome isolation methods.

Isolation methods	Basic principle	Advantages	Disadvantages	Refs.
Ultracentrifugation	Size Shape Density	Gold‐standard Low cost Low protein contamination Large sample capacity High yields	Time‐consuming High centrifugation speed leads to potential damage Costly instruments Low portability Low recovery rate	^41^, ^47^
Immunomagnetic beads	The specific interactions between the surface proteins of exosomes (antigens) and magnetic beads coated with immobilized antibodies	Time‐saving Specific separation High purity High possibility to isolate the subtypes of exosomes	Only exosomes with the target proteins can be isolated Expensive Low capacity	^44^, ^48^
Polyethylene glycol (PEG)‐based precipitation	The solubility or dispersibility	Simple and fast procedure High scalability Does not require specialized equipment Can integrate with other isolation methods	Possible contamination by non‐exosomal substances Low sample volumes	^49^
Size‐exclusion chromatography	Hydrodynamic volume	Easy High scalability Cost‐effective Does not require expensive equipment Can preserve the biological activity and integrity	Low dilution and resolution Contamination of same‐sized vesicles	^19^, ^50^
Ultrafiltration	Size Molecular weight	Simple and fast procedure Does not require special equipment High scalability Time efficiency	Low recovery rate May potentially damage the integrity	^51^
Microfluidics‐based techniques	Size Density Specific surface markers	High sensitivity High‐throughput Rapid separation Save samples Easy integration with other techniques	The need for self‐made microfluidic devices and difficult to operate	^19^, ^39^, ^44^

## 3 TDES PROMOTE LIVER METASTASIS BY FACILITATING LIVER PMN FORMATION

TDEs are exosomes released by tumour cells widely present in the tumour tissues and various body fluids of patients with tumours.^52^ Interestingly, tumour cells secrete more exosomes than normal cells do, possibly because of stress, including the hypoxia prevalent in the tumour microenvironment, which causes increased levels of exosomes in patients with tumours, particularly in those with advanced or metastatic tumours.^53^ Current studies have shown that TDE‐mediated cellular communication benefits tumorigenesis and drives tumour metastasis, and the concept of TDEs as potent mediators of intracellular interactions is now widely accepted.^25^, ^54^, ^55^

**FIGURE 2:**
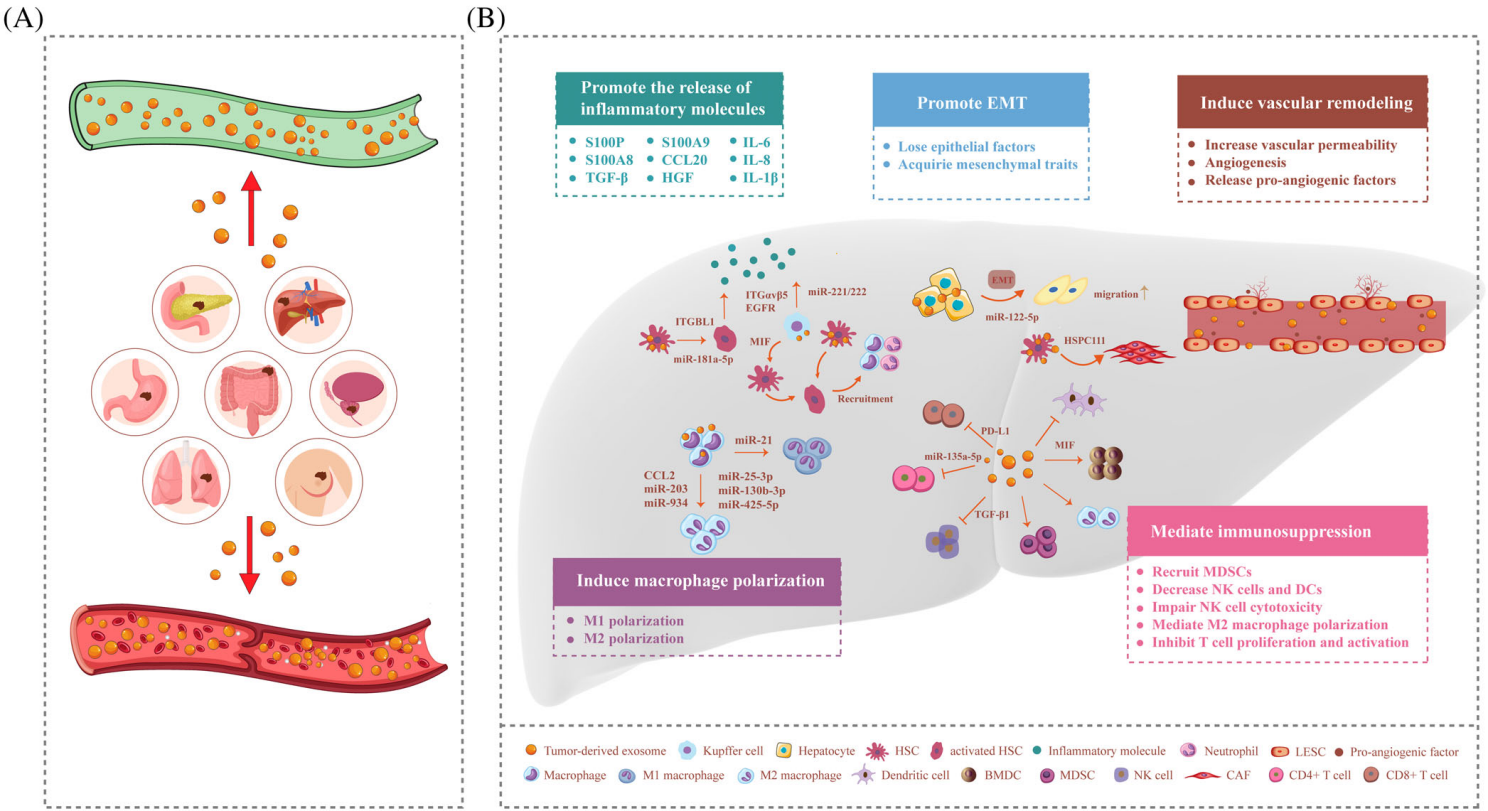
Effects of tumour‐derived exosomes (TDEs) on liver pre‐metastatic niche formation. (A) Exosomes are released by a variety of tumours, including colorectal, pancreatic, gastric, liver, breast, lung, and prostate cancers. Subsequently, TDEs reach the liver through the blood and lymphatic system to promote the formation of liver pre‐metastatic niche. (B) TDEs induce the liver pre‐metastatic niche formation by promoting the release of inflammatory molecules, epithelial–mesenchymal transition (EMT); inducing vascular remodelling, and macrophage polarization; and mediating immunosuppression.

As early as 1889, Step Paget proposed the ‘seed and soil’ theory, which indicates that tumour metastasis is not random but determined by seeds and soil that provide favourable conditions for tumour cell growth.^56^ Primary tumours induce adaptive changes in the microenvironment of distant organs to form a PMN that provides an environment needed for the colonization of circulating tumour cells and promotes the occurrence of tumour metastasis.^14^, ^57^ Interestingly, ‘seeds’ can affect the ‘soil’ via TDEs, and TDEs contribute to liver PMN formation and consequently increase the liver metastatic burden. According to current studies, the defining principles of TDE‐mediated liver PMN formation include promoting the release of inflammatory molecules, epithelial–mesenchymal transition (EMT), inducing vascular remodelling and macrophage polarization, and mediating immunosuppression (Figure 2). Here, we summarize the mechanisms by which TDEs aid liver PMN formation following the aforementioned aspects (Table 2).

**TABLE 2:** Typical studies on the roles of TDEs in liver pre‐metastatic niche formation.

Cancer type	Source	Cargos	Targeted cell	Effects and mechanism/pathway	Refs
Promote the release of inflammatory molecules					
PC	Plasma; Cell culture fluid	ITGαvβ5	KCs	Activate Src phosphorylation and upregulate the expression of S100P and S100A8	^60^
PDAC	Plasma; Cell culture fluid	MIF	KCs	Activate HSCs and TGF‐β pathways, promote fibronectin secretion, and recruit bone marrow‐derived macrophages and neutrophils	^61^
PDAC	Cell culture fluid	Not stated	Not stated	Activate HSCs, promote fibronectin secretion, increase neutrophils and macrophages, and upregulate the S100A8 and S100A9 levels	^62^
CRC	Serum; Cell culture fluid	miR‐181a‐5p	HSCs	Activate HSCs, the IL6/STAT3 signalling pathway, and the CCL20/CCR6/ERK1/2/Elk‐1/miR‐181a‐5p loop	^64^
CRC	Plasma; Cell culture fluid	ITGBL1	HSCs; S100A4^+^ fibroblasts macrophage	Induce CAFs formation by stimulating the TNFAIP3‐mediated NF‐κB pathway and produce high levels of inflammatory molecules, including IL‐6, IL‐8, and IL‐1β	^65^
CRC	Serum; Cell culture fluid	miR‐221/222	Liver stromal cells (KCs and HSCs)	Activate HGF by suppressing the SPINT1 expression	^66^
GC	Serum; Cell culture fluid	EGFR	Liver stromal cells (KCs and HSCs)	Activate HGF by directly targeting miR‐26a/b	^67^
Promote EMT and induce vascular remodelling					
LC	Cell culture fluid	miR‐122‐5p	Hepatocytes	Affect the migration of liver cells and transmission of more EMT‐related properties to normal liver cells	^70^
CRC	Serum; Cell culture fluid	ADAM17	Not stated	Increase E‐cadherin cleavage and upregulates the mesenchymal expression	^71^
CRC	Serum; Cell culture fluid	HSPC111	HSCs	Induce CAFs formation and reprograms the lipid metabolism through phosphorylation of ACLY to increase the level of acetyl‐CoA. In turn, CAFs induce EMT via the CXCL5‐CXCR2 axis	^73^
TNBC	Cell culture fluid	Not stated	LSECs	Induce endothelial to mesenchymal transition and disrupt the vascular endothelial barriers	^76^
HCC	Serum; Cell culture fluid	miR‐638, miR‐663a, miR‐3648, miR‐4258	HUVECs	Attenuate endothelial junction integrity and promote vascular permeability by inhibiting VE‐cadherin and the ZO‐1 expression	^78^
CRC	Serum; Cell culture fluid	miR‐25‐3p	HUVECs	Promote vascular permeability and angiogenesis by regulating the expression of VEGFR2, ZO‐1, occluding, and claudin5 in endothelial cells by targeting KLF2 and KLF4	^75^
GC	Serum; Cell culture fluid	miR‐519a‐3p	Macrophages	Induce the formation of angiogenesis‐rich liver PMN by inducing M2‐like polarization of macrophages by activating the DUSP2‐MAPK/ERK axis	^83^
CRC	Tissue; Cell culture fluid	ANGPTL1	KCs	Impede vascular leakiness by regulating KCs secretion pattern and decrease its MMP9 levels by inhibiting the JAK2‐STAT3 pathway	^84^
Induce macrophage polarization and mediate immunosuppression					
CRC	Serum	CCL2	Macrophages (KCs)	Activate KCs recruitment and mediate polarization of M2 macrophages	^97^
CRC	Serum; Cell culture fluid	miR‐203	Monocytes	Promote the differentiation of monocytes to M2‐TAMs	^98^
CRC	Cell culture fluid	miR‐934	Macrophages	Induce M2 macrophage polarization by downregulating the PTEN expression and activating the PI3K/AKT pathway, thus activating a CXCL13/ CXCR5/NFκB/p65/miR‐934 positive feedback loop	^99^
CRC	Serum; Cell culture fluid	miR‐25‐3p, miR‐130b‐3p, miR‐425‐5p	Macrophages	Promote M2 polarization of macrophages through regulating PTEN by activating the PI3K/AKT pathway, thereby enhancing EMT and secreting VEGF	^82^
CRC	Plasma; Cell culture fluid	miR‐21	Macrophages (KCs)	Induce M1 macrophage polarization through the miR‐21–TLR7–IL‐6 axis	^100^
BC	Cell culture fluid	Not stated	CD45^+^ bone marrow‐derived cells	Accumulation of MDSCs and decrease in NK cells	^102^
BC	Plasma; Cell culture fluid	CCL2	CCR2 positive cells	Accumulation of Ly6C^−^macrophages and decrease in DCs	^104^
PDAC	Serum; Cell culture fluid	TGF‐β1	NK cells	Inhibit NK cell function and impair NK cell cytotoxicity by the TGFβ‐Smad2/3 signalling pathway	^105^
CRC	Serum; Cell culture fluid	miR‐135a‐5p	KCs	Inhibit CD30‐mediated CD4^+^ T activation and promote CRC cell adhesion by activating the LATS2‐YAP1/TEAD1‐MMP7 pathway	^107^

*Abbreviations*: ACLY, ATP‐citrate lyase; ADAM17, A disintegrin and metalloproteinase 17; ANGPTL1, angiopoietin‐like protein 1; BC, breast cancer; CAFs, cancer‐associated fibroblasts; CCL2, CC chemokine ligand‐2; CRC, colorectal cancer; DCs, dendritic cells; DUSP2, dual specificity protein phosphatase 2; EGFR, epidermal growth factor receptor; EMT, epithelial‐mesenchymal transition; GC, gastric cancer; HCC, hepatocellular carcinoma; HGF, hepatocyte growth factor; HSCs, hepatic stellate cells; HUVECs, human umbilical vein endothelial cells; ITGαvβ5, integrin αvβ5; ITGBL1, integrin beta‐like 1; KLF2, krüppel‐like factor 2; KLF4, krüppel‐like factor 4; KCs, Kupffer cells; LATS2, large tumour suppressor kinase 2; LC, lung cancer; LSECs, liver sinusoidal endothelial cells; MDSCs, myeloid‐derived suppressor cells; MIF, macrophage migration inhibitory factor; MMP9, matrix metalloproteinase‐9; NK, natural killer; PC, pancreatic cancer; PDAC, pancreatic ductal adenocarcinoma; SPINT1, serine protease inhibitor Kunitz type; TAMs, tumour‐associated macrophages; TGF‐β1, transforming growth factor‐β1; TNBC, triple‐negative breast cancer; TNFAIP3, tumour necrosis factor alpha‐induced protein 3; 1; VE‐cadherin, vascular endothelial‐cadherin; ZO‐1, zonula occludens‐1.

### 3.1 TDEs promote the release of inflammatory molecules

Chronic inflammation affects cancer metastasis into or within the liver, creating a microenvironment for tumour cells to metastasize quickly.^58^ Classically, inflammatory molecules, including chemokines and cytokines, are the master regulators of inflammatory processes in a tumour microenvironment.^59^ Interestingly, TDEs contributed to the release of inflammatory molecules, which frequently correlated with PMN formation in the liver. Hoshino et al. found that TDEs expressing ITGαvβ5 were associated with liver‐specific metastasis. They could specifically bind to liver KCs, activate Src phosphorylation, and subsequently upregulate the expression of cell migration genes, especially pro‐inflammatory S100P and S100A8, thereby initiating an inflammatory PMN in liver‐specific metastasis.^60^ Costa Silva et al. showed that the sequential steps of pancreatic ductal adenocarcinoma (PDAC)‐derived exosomes induced the establishment of an inflammatory PMN in the liver, and they first demonstrated in a mouse model that PDAC‐derived exosomes were mainly taken up by hepatic KCs and not by other liver‐resident cells. Subsequently, they found that KCs engulfed PDAC‐derived exosomes carrying macrophage migration inhibitory factor (MIF) and could induce liver fibrosis pathways, especially transforming growth factor (TGF)‐β signalling, resulting in HSCs activation and fibronectin production. In turn, fibronectin accumulation facilitates the recruitment of bone marrow‐derived macrophages and neutrophils in the liver, creating a favourable liver PMN that allows disseminated PDAC cells to survive and grow. Importantly, depleting MIF in PDAC‐derived exosomes can prevent the formation of a liver PMN. Moreover, high exosomal MIF levels were observed in plasma from patients with PDAC with liver metastasis, suggesting that exosomal MIF is important for initiating a liver PMN and may provide a diagnostic value and therapeutic target for PDAC metastasis to the liver.^61^ Similarly, another study also confirmed that exosomes derived from highly metastatic PDAC cells could activate HSCs, produce fibronectin, increase neutrophils and macrophages, and upregulate pro‐inflammatory S100A8 and S100A9 in the liver compared with exosomes derived from weakly metastatic PDAC cells.^62^ The above studies show that S100A8 can act as a driver of liver PMN formation, suggesting that it may have potential clinical application in liver metastasis.

In addition to PDAC, the most common target organ for colorectal cancer (CRC) metastasis is the liver, with up to 30%–50% of patients developing liver metastasis during their disease course, and approximately 50% of patients have synchronous liver metastases at initial diagnosis.^63^ Recently, Zhao reported that miR‐181a‐5p was enriched in exosomes derived from highly metastatic CRC cells, which could activate HSCs by targeting the suppressor of cytokine signalling 3 (SOCS3) and activating the interleukin (IL)6/signal transducer and activator of transcription 3 (STAT3) signalling pathway. Intriguingly, activated HSCs could secrete C‐C motif chemokine ligand (CCL)20, further activating the positive feedback loop of CCL20/CCR6/ERK1/2/Elk‐1/miR‐181a‐5p to remodel the liver PMN and promote liver metastasis. Furthermore, miR‐181a‐5p expression in serum exosomes from patients with CRC with liver metastasis was significantly upregulated.^64^ Ji et al. also showed that CRC cell‐derived integrin beta‐like 1 (ITGBL1)‐enriched exosomes were directly transferred to the liver, and they activated HSCs by binding to tumour necrosis factor alpha‐induced protein 3 (TNFAIP3) and then activated the nuclear factor kappa light chain enhancer of activated B cells (NF‐κB) signalling pathway, accompanied by the secretion of high levels of cytokines, such as IL‐6 and IL‐8, to create a fibrotic PMN and promote CRC stemness, aggressiveness, EMT, and liver metastasis.^65^ Additionally, CRC‐derived exosomes harbouring miR‐221/222 induced liver stromal cells to secret hepatocyte growth factors (HGFs) by suppressing serine protease inhibitor Kunitz type 1 (SPINT1) expression, providing a hospitable liver PMN for the aggressiveness of CRC cells.^66^ Consistently, gastric cancer (GC) cell‐derived exosomal epidermal growth factor receptor (EGFR) can integrate with liver stromal cells and can effectively activate HGFs by directly targeting miR‐26a/b, preparing fertile ‘soil’ for future gastric cell metastasis to the liver.^67^ These studies suggest that TDEs are critical tumour‐secreted factors involved in the release of pro‐inflammatory factors for facilitating the establishment of a liver PMN.

### 3.2 TDEs promote EMT

EMT is a key process in the initiation of tumour metastasis.^68^, ^69^ In this process, molecular changes are often accompanied, such as the loss of epithelial markers and the gain of mesenchymal markers.^13^ Recent studies suggest that TDEs are important in liver metastasis to facilitate EMT in normal liver cells and cancer cells. In lung cancer, hnRNPA2B1 mediates the packaging of miR‐122‐5p into EVs as exosomes, and exosomal miR‐122‐5p imparts more EMT‐related properties to hepatocytes, including increasing the levels of the mesenchymal markers N‐cadherin and vimentin and decreasing the level of epithelial marker E‐cadherin, as well as promoting the migration of hepatocytes, thus promoting PMN formation suitable for the seeding of metastatic cancer cells.^70^ In addition, a recent study showed that exosomes from CRC cells containing high levels of functional A disintegrin and metalloproteinase 17 (ADAM17) increased the levels of N‐cadherin, vimentin, and snail, as well as E‐cadherin cleavage, promoting EMT in CRC cells, thus promoting CRC cell dissemination from the primary lesion to the liver.^71^

TDEs can not only modify normal liver cells or cancer cells to acquire a mesenchymal phenotype but also induce the formation of cancer‐associated fibroblasts (CAFs)^55^, ^56^; CAFs can produce exosomes, which in turn stimulate EMT and reprogramme the tumour microenvironment.^72^ For example, CRC cell‐derived exosomal HSPC111 could convert HSCs to CAFs and reprogram lipid metabolism by phosphorylating ATP‐citrate lyase to increase acetyl‐CoA levels in these CAFs, resulting in liver PMN formation. Moreover, CAFs induced the EMT and liver metastasis of CRC cells via the C‐X‐C motif chemokine ligand (CXCL)5‐C‐X‐C chemokine receptor (CXCR)2 axis, as indicated by increasing vimentin and snail levels and decreasing E‐cadherin levels.^73^ Interestingly, another study also showed that CAF‐secreted exosomal miR‐92a‐3p could evoke EMT, enhance stemness, and inhibit apoptosis in CRC cells, leading to liver metastasis and chemotherapy resistance.^74^ These observations highlight an important effect of TDEs on EMT development and CAF formation; thus, deciphering the mechanisms of TDE‐mediated EMT in a liver PMN is essential.

### 3.3 TDEs induce vascular remodelling

Increasing vascular permeability and promoting angiogenesis are also crucial in PMN formation.^15^ Compelling evidence shows that TDEs are critical modulators of vascular remodelling by the functional reprogramming and phenotypic modulation of endothelial cells, which may be closely related to the establishment of a liver PMN and the enhancement of the metastatic liver burden in various cancers.^75^, ^76^

GC cell‐derived EVs containing AMIGO2 significantly enhanced the adhesion of LSECs to cancer cells and promoted the initial step of liver metastasis.^77^ Using an in vitro three‐dimensional microfluidic human liver‐on‐a‐chip, liver PMN formation was reconstructed, and Kim et al. reported that breast cancer‐derived EVs could activate LSECs and increase fibronectin on LSECs, inducing endothelial to mesenchymal transition and disrupting vascular endothelial barriers.^76^ Highly intrahepatic metastatic hepatocellular carcinoma (HCC)‐cell‐derived exosomal miRNAs, including miR‐638, miR‐663a, miR‐3648, and miR‐4258, attenuated endothelial junction integrity and promoted vascular permeability by decreasing the endothelial expression of vascular endothelial‐cadherin (VE‐cadherin) and zonula occludens‐1 (ZO‐1), thus initiating liver PMN formation.^78^ In addition, HCC cell‐secreted exosomal miR‐103 could also be delivered to endothelial cells and increased vascular permeability by directly inhibiting multiple endothelial junction proteins, including VE‐cadherin, p120‐catenin, and ZO‐1, which facilitated the transendothelial invasion of cancer cells and liver metastasis.^79^ Similarly, in CRC, exosomal miR‐25‐3p was delivered to endothelial cells, which increased vascular endothelial growth factor (VEGF) receptor 2 levels and decreased ZO‐1, occluding, and claudin5 levels by targeting krüppel‐like factor (KLF) 2 and KLF4, thus promoting vascular permeability and angiogenesis *in vivo* and inducing liver PMN formation.^75^ Strikingly, pancreatic cancer cell‐derived exosomal circ‐IARS accessed human microvascular vein endothelial cells, significantly inhibited miR‐122 and ZO‐1 levels, and increased RhoA and F‐actin levels and focal adhesion, ultimately increasing vascular endothelial permeability and tumour metastasis.^80^ Besides the affected phenotypic characteristics of endothelial cells, TDEs directly regulated the release of pro‐angiogenic factors, such as VEGF, MMP2, MMP9, basic fibroblast growth factor, and TGF‐β, further promoting angiogenesis and tumour progression.^81^, ^82^ Additionally, a novel studies by Qiu et al. demonstrated that TDEs could create a vascular‐rich liver PMN by acting on macrophages. They revealed that GC‐derived exosomal miR‐519a‐3p could induce M2‐like polarization of macrophages by activating the dual specificity protein phosphatase 2‐MAPK/ERK axis, thereby inducing the formation of angiogenesis‐rich liver PMN and facilitating liver metastasis.^83^ However, more evidence is needed to support TDEs‐mediated angiogenesis by targeting macrophages.

Conversely, TDEs can also suppress angiogenesis in liver metastasis. In a study by Jiang et al., CRC‐derived exosomes containing ANGPTL1 proteins attenuated liver metastasis and impeded vascular leakiness in a liver PMN by regulating KCs secretion pattern and decreasing its MMP9 levels by inhibiting the Janus kinase (JAK2)‐STAT3 signalling pathway; however, whether exosomal ANGPTL1 affected vascular permeability by directly or indirectly affecting endothelial cells remained unclear.^84^ Another study also showed that exosomal circFNDC3B inhibited CRC angiogenesis and liver metastasis by regulating the circFNDC3B‐miR‐97‐5p‐TIMP3 pathway.^85^ Altogether, these data showed that TDEs were heterogeneous, and TDE‐induced vascular remodelling depended on molecular and genetic cargos that TDEs delivered to recipient cells. Further evidence on the mechanisms of how TDEs shape a liver PMN via vascular remodelling is necessary. Moreover, a deeper understanding of TDE‐driven vascular remodelling will improve anti‐angiogenic therapies targeting TDEs in the future.

### 3.4 TDEs induce macrophage polarization

Macrophages are an indispensable part of the liver microenvironment, and it contains resident KCs and infiltrating monocytes, which play an important role in regulating liver inflammation, HSC activation, and fibrosis.^86^, ^87^, ^88^, ^89^ Macrophages exhibit strong plasticity and can change their phenotypes in response to microenvironmental signals. Based on the activation status and functions, macrophages are mainly classified into M1 (pro‐inflammatory) and M2 (immune‐suppressive) phenotypes.^90^, ^91^ Recent studies showed that TDEs are closely associated with macrophage polarization,^92^, ^93^, ^94^, ^95^ and TDE‐mediated macrophage phenotypic changes are key mediators during liver PMN establishment and maintenance.^92^, ^93^, ^96^

In CRC, exosomes carrying CCL2 prime the formation of fibrotic and suppressive liver PMN by activating macrophage (KCs) recruitment and shifting the M1/M2 paradigm to the M2 phenotype. Furthermore, the authors found that Dahuang Zhechong Pill can inhibit liver metastasis in CRC by reducing macrophage infiltration and TGF‐β1 gene expression, suppressing the CCL2‐mediated polarization of M2 macrophages.^97^ Another study showed that CRC‐derived exosomes carrying miR‐203 were incorporated into monocytes, which showed a significant increase in the M2 markers (CD163 and STAT3) of macrophages co‐cultured with CRC‐derived exosomes.^98^ Zhao et al. revealed an important mechanism of the CRC‐derived exosome‐mediated polarization of macrophages. The authors first found that the miR‐934 level was significantly increased in the tissue and serum samples of patients with CRC with liver metastasis, and miR‐934 was an independent prognostic biomarker of liver metastasis in CRC. Subsequently, they found that hnRNPA2B1 mediated the packaging of miR‐934 into the exosomes of CRC cells and then delivered exosomal miR‐934 into macrophages to induce the polarization of M2‐type macrophages. They also demonstrated that exosomal miR‐934 induced M2 macrophage polarization via downregulating PTEN expression and activating the phosphoinositide‐3‐kinase (PI3K)/AKT signalling pathway, and polarized M2 macrophages induced liver PMN formation and promoted liver metastasis in CRC by activating the CXCL13/CXCR5/NFκB/p65/miR‐934 positive feedback loop.^99^ In particular, CRC cell‐derived exosomal miR‐25‐3p, miR‐130b‐3p, and miR‐425‐5p could also be transferred into macrophages to promote M2 polarization by regulating PTEN via activating the PI3K/AKT signalling pathway. Subsequently, M2‐polarized macrophages promoted liver PMN formation and liver metastasis by enhancing EMT and secreting VEGF.^82^ Based on the above important findings, TDEs are considered cellular messengers to promote the differentiation of macrophages to the M2 phenotype, thus creating a favourable PMN for liver metastasis.

Although most studies have shown that TDEs can polarize macrophages into the M2 phenotype, Shao et al. showed that TDEs could potentially orchestrate M1‐phenotype macrophage polarization. They reported that CRC‐derived exosomes carrying miR‐21 were specifically colocalized with KCs. These KCs were polarized to the IL‐6‐secreting M1 phenotype via the toll‐like receptor (TLR)7 pathway, which created a permissive inflammatory PMN in the mouse liver, thereby promoting the survival and colonization of CRC cells in the liver and eventually led to liver‐specific metastasis. Moreover, miR‐21 levels in exosomes from the plasma samples of patients with CRC with liver metastasis were significantly increased, and plasma IL‐6 levels were also observed significantly higher in CRC patients with liver metastasis.^100^ These studies show that macrophages are critical for receiving and transmitting tumour exosome messages for promoting liver organotropic metastasis. Therefore, exploring the mechanisms of TDE‐induced macrophage polarization and the role of M1/M2 polarized macrophages in promoting liver metastasis is necessary.

### 3.5 TDEs mediate immunosuppression

TDEs also modulate immune responses, an important step in liver PMN establishment. TDEs could promote the recruitment of immune‐suppressing cells, which skewed the immune repertoire toward an immunosuppressive kind. In PDAC, TDEs promoted bone marrow‐derived macrophage recruitment in liver PMN.^61^, ^62^ Bone marrow‐derived cell‐secreted EV miR‐92a could specifically promote lung cancer metastasis to the liver by potentiating HSCs activation and increased extracellular matrix deposition, which promoted the recruitment of MDSCs in the liver and fostered the formation of an immunosuppressive PMN for liver metastasis.^101^ In a study on the effects of exosomes derived from breast cancer cells on liver metastasis, highly metastatic breast cancer cell‐derived exosomes contributed to MDSCs accumulation in the liver, accompanied by decreased NK cells, thus constructing a favourable immunosuppressive PMN permissive to metastatic colonization in the liver.^102^ Strikingly, in oesophageal squamous cell carcinoma, local irradiation could change the composition of exosomes, promote the accumulation of MDSCs, and enhance their immunosuppressive function by activating the PI3K/AKT pathway, resulting in the formation of a metastasis‐promoting microenvironment.^103^ Of note, TDEs can associate with tumour‐microenvironmental cytokines, including CCL2 and IL‐6, and can subsequently induce changes in the immune microenvironment landscape of the liver, manifested as a lower DC frequency and a higher macrophage frequency.^104^ Additionally, as mentioned above, TDEs are essential mediators of modulating macrophage polarization to TAMs that display the M2 phenotype in a liver PMN, thus providing an immunosuppressive liver microenvironment for cancer cell growth and invasion. These observations indicated that the homing of immunosuppressive cells to the liver is a centrally important aspect of PMN formation. Inhibiting the accumulation of these cells by targeting TDEs will be a viable strategy.

On the contrary, TDEs triggered the formation of an immunosuppressive PMN of the liver by affecting immune cell functions. PDAC‐derived exosomal TGF‐β1 inhibited the functions of NK cells by downregulating the activating receptors (NKG2D and CD107a), decreasing cytokine release (TNF‐α and INF‐γ) and glucose uptake ability, and inducing the phosphorylation of Smad2/3 in NK cells. Furthermore, NK cells pretreated with PDAC‐derived exosomes showed the attenuation of NK cell cytotoxicity against pancreatic cancer stem cells, which allowed metastatic tumour cells to escape from NK cell immune surveillance in the liver PMN.^105^ Recent studies have also shown that TDEs affect T cell function. Lundholm et al. showed that prostate TDEs downregulated the expression of NKG2D on NK and CD8^+^ T cells, which resulted in impaired cytotoxic function and tumour immune escape.^106^ Sun et al. discovered that exosomal miR‐135a‐5p promoted CRC cell adhesion by activating the LATS2‐YAP1/TEAD1‐MMP7 pathway and inhibited CD30‐mediated CD4^+^ T activation, thereby selectively facilitating the formation of an immunosuppressive PMN for CRC liver metastases (CRLM). Of note, the researchers have also observed that hypoxia could not only promote exosome secretion but also increase the expression of exosomal miR‐135a‐5p.^107^ Therefore, it will be interesting and meaningful to further investigate the regulation of liver metastasis by exosomes under hypoxic conditions. In addition, TDEs carring PD‐L1 could inhibit the proliferation of CD8^+^ T cells,^16^, ^108^ and exosomal PD‐L1 was negatively associated with T lymphocyte infiltration and activation in liver metastasis,^109^ which suggested that exosomal PD‐L1 contributes to immunosuppression and promotes the development of liver PMN. These findings supported that TDEs are responsible for the formation of liver immunosuppressive PMN, consequently promoting liver metastasis, which can yield novel strategies to inhibit liver metastasis. However, immune therapy based on TDEs is still in the early stage; it will certainly be critical for future work to investigate their efficiency and further explore how TDEs collaborate to regulate the liver PMN by mediating immunosuppression.

## 4 CLINICAL APPLICATIONS

### 4.1 TDEs as diagnostic and prognostic biomarkers in liver metastasis

Many previous studies have shown that exosomes have great potential as novel biomarkers in liquid biopsy, which have the following significant advantages^30^, ^50^, ^110^, ^111^: (1) They can decrease the risk of the deterioration of disease caused by some invasive biopsies. (2) They are easy to operate and do not require imaging support. (3) Samples of body fluids are easier to collect and their experiments are replicable, which is more conducive to the dynamic monitoring of tumour progression. (4) It can effectively deal with tumour heterogeneity caused by tissue biopsy. (5) It has certain advantages in the early diagnosis of tumours, and the detection of tumours can be earlier than imaging detection. In 2016, the first exosome‐based cancer diagnostic tool was launched in the United States of America.^112^ To date, miRNA and proteins are the most widely studied exosomal cargo as diagnostic and prognostic biomarkers for liver metastasis (Table 3).

**TABLE 3:** Exosomal miRNAs and proteins as potential biomarkers in liver metastasis.

Biomarker type	Cancer types	Exosome sources	Exosomal biomarkers	Exosome isolation method	Exosome detection method	Selection cohort	Validation cohort	Clinical significance	Refs
Diagnostic	CRC	Plasma	miR‐21	UC	miRNA microarray; qRT‐PCR	3 CRC patients; 3 HCs	326 CRC patients; 30 HCs	Higher levels associated with liver metastasis and TNM stage	^113^
Diagnostic and prognostic	CRC	Serum	miR‐122	Exosome isolation kit	qRT‐PCR	12 CRLM patients; 12 CRC patients without LM; 12 HCs	35 CRLM patients; 50 CRC patients without LM; 50 HCs	Distinguish CRLM patients from patients without LM and HCs; Higher levels associated with poor OS	^114^
Diagnostic	CRC	Plasma	miR‐150	Exosome isolation kit	miRNA arrays; qRT‐PCR	10 CRLM patients; 10 CRC patients without metastases; 10 HCs	64 CRC patients	Lower levels related to LM and TNM stage	^115^
Diagnostic	CRC	Serum	miR‐548c‐5p	Exosome isolation kit	qRT‐PCR	108 CRC patients		Lower levels associated with LM and later TNM stage	^116^
Diagnostic	CRC	Serum	miR‐638	Exosome isolation kit	miRNA arrays; qRT‐PCR	3 CRC patients; 3 HCs	77 CRC patients; 20 HCs	Lower levels associated with LM and later TNM stage	^117^
Diagnostic	CRC	Serum	miR‐6803‐5p	Exosome isolation kit	miRNA arrays; qRT‐PCR	168 CRC patients; 20 HCs		Higher levels associated with LM, LNM, and later TNM stage	^118^
Diagnostic	CRC	Serum	miR‐ 221/222	Exosome isolation kit	qRT‐PCR	40 CRLM patients; 40 CRC patients without metastases; 40 HCs		Higher levels in CRLM patients	^66^
Prognostic and predictive	CRC	Plasma; Serum	miR‐6087, miR‐132‐5p, miR‐93‐3p, miR‐320d	Exosome isolation kit	RNA sequencing; qRT‐PCR	6 paired nonmetastatic CRC and CRLM patients; 10 nonmetastatic CRC and 20 CRLM patients	Training: 113 CRLM patients; Internal:114 CRLM patients; External:168 CRLM patients;	Negatively correlated with the RFS and OS; Patients with 4 exosomal miRNA‐classifier‐defined high‐risk scores benefited from adjuvant chemotherapy	^119^
Diagnostic	RC	Plasma	miR‐141‐3p and miR‐375	Exosome isolation kit	miRNA microarray; qRT‐PCR	29 RC patients	64 RC patients	Differentiate patients with LM from patients without LM	^120^
Diagnostic and prognostic	GC	Plasma	miR‐151a‐3p	Polymeric precipitation, UF and UC	miRNA sequencing; qRT‐PCR	5 GC‐LM patients; 5 GC patients without LM	30 GC‐LM patients and 30 GC patients without LM; 50 GC‐LM, 50 GC patients without LM, and 30 HCs	Differentiate GC‐LM patients from patients without LM and HC; Negatively correlated with the OS of GC‐LM patients	^121^
Diagnostic	GC	Plasma	miR‐143‐5p	Exosome isolation kit	RNA sequencing; qRT‐PCR	18 GC patients (4 primary GC, 4 GC with LNM, 4 GC with OM, 6 GC with LM); 5 non‐cancerous	108 GC patients (30 primary GC, 33 GC with LNM, 14 GC with OM, 31 GC with LM); 23 non‐cancerous	Distinguish GC patients with LM from primary GC patients	^122^
Diagnostic	GC	Plasma	miR‐200c‐3p and miR‐429	SEC	RNA sequencing; qRT‐PCR	40 GC patients, including M0, PM, LM, and dLNM	Training: 40 GC patients; Validating: 86 GC patients	Distinguish LM and relevant organotropism	^123^
Prognostic	CRC	Plasma	PD‐L1	ExoQuick™	ELISA	49 CRLM patients		Preoperative levels were negatively correlated with RFS and OS; Higher postoperative levels were associated with a higher early recurrence rate.	^109^
Prognostic	CRC	Serum	TIMP‐1	Exo‐spin™ exosome purification kit	ELISA	151 CRLM patients	Internal: 49 CRLM patients; External: 56 CRLM patients	Higher levels associated with a shortened OS	^124^
Prognostic	CRC	Serum	CD14, LBP, Serpin A4 and CFP	Not stated	Mass spectrometry; ELISA	56 CRLM patients; 7 patients with BD	Internal: 154 CRLM patients; 78 patients with BD; External: 110 CRLM patients	Negatively correlated with the OS	^125^
Prognostic	PDAC	Tissue and serum	CD44v6 and C1QBP	Tissue: UC and UF serum: Exosome isolation kit	Western blot	Tissue: normal pancreas; PDAC with LM and without LM; Serum: 20 patients with LM; 122 patients without LM		Higher levels associated with worse OS	^126^

*Abbreviations*: BD, benign liver disease; CRC, colorectal cancer; CRLM, colorectal liver metastasis; dLNM, distant lymph node metastasis; GC, gastric cancer; HCs, healthy controls; LM, liver metastasis; LNM, lymph node metastasis; M0, no metastasis; OM, ovarian metastasis; OS, overall survival; PDAC, pancreatic ductal adenocarcinoma; PM, peritoneal metastasis; RFS, recurrence‐free survival; RC, rectal cancer; SEC, size exclusion chromatography; UC, ultracentrifugation; UF, ultrafiltration.

#### 4.1.1 Tumour‐derived exosomal miRNAs

In patients with CRC, the levels of miR‐21 in plasma exosomes were significantly higher than those of healthy individuals, and elevated exosomal miR‐21 expression was significantly associated with liver metastasis and TNM stage, suggesting that exosomal miR‐21 may be a promising non‐invasive diagnostic biomarker for patients with CRLM.^113^ Evaluation of the diagnostic and prognostic value of exosomal miR‐122 levels in patients with CRC has been documented. Patients, especially those with liver metastasis, with a high concentration of exosomal miR‐122 have diminished overall survival (OS). Receiver‐operating curve (ROC) analysis also showed that serum exosomal miR‐122 could differentiate patients with CRC with liver metastasis from patients without liver metastasis and healthy individuals with an area under the ROC curve (AUC) of 0.81 and 0.89.^114^ Other studies also showed that miR‐150, miR‐638, and miR‐548c‐5p were downregulated in the serum exosomes of patients with liver metastases compared with those of patients without liver metastasis,^115^, ^116^, ^117^ whereas exosomal miR‐6803‐5p were significantly higher in the serum of patients with CRC with advanced TNM or lymph node metastasis and liver metastasis, and higher levels of exosomal miR‐6803‐5p were linked with poorer survival outcomes.^118^ In serum exosomes from 40 patients with CRLM, 40 patients with CRC without metastasis, and 40 normal subjects, Tian et al. found that exosomal miR‐221/222 were significantly enriched in the serum of patients with CRLM, and high levels of miR‐221/222 indicated poor progression‐free interval rate.^66^ In addition, by using miRNA sequencing and the LASSO model, Wang et al. developed a predictive model based on pre‐hepatectomy circulating exosomal miRNA signatures. As expected, the model showed good performance in predicting the prognosis and adjuvant chemotherapy benefit in patients with CRLM following hepatectomy.^119^ This presents exosomal miRNA signatures as a promising prognostic biomarker and guides adjuvant chemotherapy decisions for patients with CRLM the following hepatectomy.

In patients with rectum cancer, plasma exosomal miR‐141‐3p and miR‐375 were highly expressed in patients with liver metastasis than those without liver metastasis and could be reliable biomarkers for diagnosing liver metastasis.^120^ MiR‐151a‐3p is highly expressed in plasma‐derived exosomes of patients with GC with liver metastasis compared with patients with non‐liver metastasis and healthy volunteers, which is a useful diagnostic biomarker to identify liver metastasis, with an AUC of 0.707. Moreover, the expression of plasma exosomal miR‐151a‐3p was negatively correlated with the outcome of patients with GC and liver metastasis, which indicated its potential as a candidate prognostic biomarker.^121^ Another example of exosomal miRNA as a diagnostic biomarker is exosomal miR‐143‐5p in the plasma of patients with GC and liver metastasis, which has 70.97% sensitivity and 86.67% specificity.^122^ In addition, Zhang et al. analysed plasma exosomal miRNA from patients with GC and peritoneal metastasis, liver metastasis, distant lymph node metastasis, and without metastasis, aiming to identify exosomal miRNA signatures that could predict metastatic organotropism of GC. Their results showed that plasma exosomal‐miRNA signatures characterized and predicted the organo‐tropic metastasis of GC. Among them, the combination of exosomal miR‐200c‐3p and miR‐429 can effectively characterize liver metastasis of GC, which provided new insights into the applications of exosomal cargos in liver metastasis diagnostics and prognostics.^123^

#### 4.1.2 Tumour‐derived exosomal proteins

As exosomal miRNA is an ideal candidate for non‐invasive liquid biopsy and early detection, there is a growing interest in exosomal proteins as a therapeutic tool for liver metastasis. Chen et al. reported that higher levels of exosomal PD‐L1 were associated with more significant liver metastasis lesions. Furthermore, in patients with CRLM following hepatectomy, those with higher level postoperative exosomal PD‐L1 have a high rate of early recurrence, and preoperative exosomal PD‐L1 was negatively correlated with recurrence‐free survival (RFS) and OS, which implied that exosomal PD‐L1 was a useful biomarker for the prediction of poor prognosis and early recurrence in patients with CRLM following hepatectomy.^109^ Likewise, the high levels of serum‐derived exosomal tissue inhibitor of matrix metalloproteinase‐1 (TIMP1) were associated with a dismal OS in patients with CRLM, and it is an independent biomarker for prognostic preoperative molecular stratification in patients with CRLM.^124^ Recently, Lin et al. collected matched preoperative and postoperative serum samples from patients undergoing CRLM resection and used mass spectrometry to determine exosomal proteomic profiling. They identified a signature of four exosomal proteins (CD14, LBP, Serpin A4, and CFP) that can be used as an independent prognostic biomarker to distinguish relapse and death risk in patients with CRLM.^125^ Moreover, in PDAC, many TDE proteins exerted powerful efficacy in distinguishing between patients with liver metastasis and non‐liver metastasis. Xie et al. showed that both tissues‐derived and serum‐derived exosomal CD44 variant isoform 6 (CD44v6) and complement C1q binding protein (C1QBP), were significantly increased in patients with PDAC with liver metastasis than those without liver metastasis. Compared with other patients, PDAC patients with high expression of CD44v6 and C1QBP in the tissues or serum‐derived exosomes were more likely to develop liver metastasis and had significantly worse survival outcomes, which indicated that exosomal CD44v6 and C1QBP can hold great promise for predicting prognosis and liver metastasis.^126^ Exosomal ITGαv and ITGβ5 can also be used to predict liver‐specific metastasis.^60^ Previous studies have shown that the high expression of exosomal ITGβ5 is significantly associated with liver metastasis rather than other organotropic metastases in patients with HER2‐positive GC,^127^ which provided the preliminary possibility for exosomal integrin protein as a specific biomarker for liver metastasis.

In addition to miRNAs and proteins, a recent study also showed that exosomal mRNA emerges as a promising biomarker candidate for liver metastasis. Lee et al. reported that CXCL10 RNA was highly expressed in plasma exosomes from patients with CRC with liver metastasis. Furthermore, high expression of CXCL10 was related to a poor prognosis, suggesting that CRC‐derived exosomal CXCL10 may be a novel biomarker for patients with CRLM, but more work is needed to verify its suitability as a biomarker for liver metastasis.^128^ Altogether, these findings presented TDEs as a promising diagnostic and prognostic tool in liver metastatic disease. The sensitivity and specificity of using TDEs as biomarkers also need further exploration.

### 4.2 TDEs as therapeutic targets and drug delivery systems for liver metastasis

To date, available conventional therapies for cancer are not effective for the treatment of liver metastasis.^6^, ^129^ Considering that TDEs can regulate cellular communication in the liver microenvironment by transporting various natural functional proteins and nucleic acid, studies have investigated and provided novel insights into the use of TDEs as potential therapeutic targets for liver metastatic diseases. Targeting exosome synthesis, secretion, and uptake is a promising approach to inhibiting liver metastasis. Jianpi Jiedu Recipe can reduce the secretion of CRC cells derived exosomal ITGBL1 and inhibit CRC liver metastasis by blocking the fibroblast activation by regulating the ITGBL1‐TNFAIP3‐NF‐κB signalling axis.^130^ Targeting exosomal ITGαvβ5 can also effectively decrease exosome uptake and liver‐specific metastasis.^60^ Furthermore, Park et al. showed that P2X purinoreceptor 7 (P2X7) activity directly modulates the production of breast cancer‐derived exosomes, and inhibiting P2X7 can prevent the migration of cancer cells and liver metastasis in tamoxifen‐resistant breast cancer.^131^ Major vault protein (MVP) can transport miR‐193a from colon cancer cells to exosomes and promote tumour progression. The knockout of MVPs may prevent exosome release, which will result in the accumulation of miR‐193a in tumour cells instead of exosomes and inhibit liver metastasis of colon cancer.^132^ Therefore, these findings suggested that the blockage of TDE production, secretion, and uptake may be an alternative therapeutic strategy for liver metastasis.

The ablation of exosomal‐specific active cargos provides an effective treatment option for inhibiting liver metastasis. For instance, breast cancer‐secreted exosomal miR‐4443 induced liver metastasis by downregulating the expression of tissue inhibitors of metalloproteinase 2 (TIMP2) and upregulating the expression of matrix metalloproteinases. Interestingly, when the researcher armed breast cancer‐secreted exosomes with miR‐4443 inhibitors, the liver metastasis was inhibited, which suggested that directly targeting exosomal miR‐4443 can be a valuable therapeutic modality for liver metastasis.^133^ PDAC‐derived exosomal Ezrin (EZR) promoted PDAC liver metastasis by modulating macrophage polarization into immune‐suppressive M2 phenotype, and EZR knockdown in PDAC‐derived exosomes attenuated the potential of polarizing macrophages into M2 phenotype, which was accompanied with a decrease in the degree of liver metastasis in the PDAC animal model,^96^ suggesting that exosomal EZR may be another potential therapeutic target to combat liver metastatic disease. Moreover, the therapy method targeting exosomal MIF,^61^ and miR‐21^100^ can prevent liver PMN formation and block exosome‐derived liver metastasis. These studies show that interference of exosomal specific cargos has emerged as an alternative modality and can open novel strategies for the treatment of liver metastasis.

Apart from emerging as potential drug targets, over the last few years, many studies have shown that exosomes are ideal drug delivery vehicles in cancer therapy because of their biological properties such as nanoscale size, excellent biocompatibility, high stability, low immunogenicity, low toxicity, biological barrier permeability, targeted delivery, and easy modification.^134^ More recently, Zhao et al. constructed a self‐biomimetic drug delivery system based on PDAC‐derived exosomes (PF@PCCEs) to inhibit the formation of fibrotic liver PMN and liver metastasis of PDAC by delivering an antifibrotic drug (pirfenidone, PF). They demonstrated in vitro and in vivo that PF@PCCEs were internalized by HSCs and subsequently inhibit HSCs activation, thereby exerting antifibrotic and antimetastatic effects.^135^ This study provides new data support for the possibility of treating liver metastases with TDEs‐based drug delivery systems.

ExoASO‐STAT6 is a novel engineered exosome that is designed to deliver an antisense oligonucleotide (ASO) to selectively silence the STAT6 signalling pathway in TAMs. In many preclinical tumour models, ExoASO‐STAT6 treatment resulted in an effective conversation TAMs to a pro‐inflammatory M1 phenotype, accompanied by the generation of a CD8 T cell‐mediated adaptive immune response. After intravenous administration of ExoASO‐STAT6, maximal biodistribution and STAT6‐silencing activity were observed in the liver compared with other tissues, and KCs cells displayed significantly higher ASO signals than other cell subsets. Moreover, in the HCC model, ExoASO‐STAT6 showed significant antitumoral efficacy, which suggested that tumours located in the liver, including primary liver cancer or liver metastasis of other tumour types, can benefit from treatment with ExoASO‐STAT6.^136^ Currently, a phase I clinical trial is underway to determine the efficacy, safety, and biomarkers of ExoASO‐STAT6 in patients with advanced HCC, GC, and CRC with liver metastases. However, preliminary data are not yet available and worth following up on. In addition, exosomes from mesenchymal stem cells (MSC) have been shown to inhibit breast cancer angiogenesis through VEGF downregulation in vitro and in vivo^137^ and also promote NKT‐cell antitumor response, thus suppressing HCC growth.^138^ Similarly to ExoASO‐STAT6, MSC‐derived exosomes exhibited a significantly higher biodistribution in the liver^139^ and could alleviate liver inflammation and collagen deposition by inhibiting EMT and protecting hepatocytes, thus relieving liver fibrosis.^140^ Li et al. recently showed that engineered bone marrow MSC‐derived exosomes that were transfected with small interfering RNA against GRP78 combined with Sorafenib also inhibit the growth and invasion of HCC in vitro and liver metastasis of HCC in vivo,^141^ which suggested that MSC‐derived exosomes hold the promise of emerging as an efficient delivery system in liver metastatic disease. Notably, in diverse HCC mouse models, engineered dendritic cell‐derived exosomes (DEXs) can elicit a specific antitumor immune response and tumour suppression.^142^ However, whether DEX can be used for the treatment of liver metastatic diseases is still unknown and requires further study.

## 5 CONCLUSIONS AND FUTURE PERSPECTIVES

In the present review, we elaborate on the roles and underlying mechanisms of TDEs in liver PMN formation. Exosomes derived from different tumour cells drive liver PMN formation and consequently increase liver metastatic burden by promoting EMT and the release of inflammatory factors, inducing vascular remodelling and macrophage polarization, and mediating immunosuppression. Moreover, TDEs are expected to become diagnostic and prognostic biomarkers and can be useful as novel targets and drug delivery vehicles for cancer therapy and the inhibition of liver metastasis.

Even though the vital role of TDEs in tumour metastasis of liver has been gradually shown and its application prospect is positive, it is worth noting that several challenges are yet to be addressed. The separation methods of TDEs are complicated, and no optimal separation method can ensure the purity, concentration, and activity of TDEs, which greatly limits the clinical application of TDEs. Moreover, the detection system of TDEs is not mature, which is difficult to meet the detection of large clinical samples. Therefore, more convenient, high‐throughput, and highly sensitive methods are required to optimize the extraction and detection of TDEs in the future. The function, exact delivery mechanism, and contribution rate of TDEs in the liver PMN have not been fully elucidated. Furthermore, the contents of TDEs are complex and diverse, and it is not clear whether there is a potential link among TDE contents that can promote liver metastasis. Presently, most studies on the mechanism of TDEs in liver metastasis are focused on the premetastatic stage, and the research on the metastasis stage is very limited. Interestingly, tumour metastasis is a continuous multi‐step process, and TDEs can also play crucial roles in metastasis after tumour colonization in the liver, which is worthy of further studies in the future. TDEs have great potential as biomarkers for the diagnosis and prognostic assessment of liver metastases. However, previous studies usually used small samples and had poor repeatability, which needs to be further validated in large multicenter studies to improve the reliability of liquid biopsy. Finally, targeting exosomes associated with liver metastasis is a new way to develop novel and effective anti‐tumour therapeutic agents and has the potential to become an integral part of personalized medicine. Nevertheless, little information is currently available on the involvement of exosomes in the treatment of liver metastatic disease, and establishing exosomes with satisfactory cancer‐targeting ability is highly challenging as well as large‐scale production and storage. Therefore, solving the above issues will help elucidate the underlying mechanisms of liver metastases and provide more powerful guidance for the diagnosis and treatment of liver metastasis in the future.

## AUTHOR CONTRIBUTIONS

Hua Bai and Jie Wang designed this study. Sini Li, Yan Qu, and Lihui Liu drafted the manuscript and completed the figures. Chao Wang and Li Yuan collected the references and completed the tables. All authors approved the final version of the manuscript for submission.

## CONFLICT OF INTEREST STATEMENT

The authors declare no conflicts of interest.
